# Language processing skills linked to *FMR1* variation: A study of gaze-language coordination during rapid automatized naming among women with the *FMR1* premutation

**DOI:** 10.1371/journal.pone.0219924

**Published:** 2019-07-26

**Authors:** Kritika Nayar, Walker McKinney, Abigail L. Hogan, Gary E. Martin, Chelsea La Valle, Kevin Sharp, Elizabeth Berry-Kravis, Elizabeth S. Norton, Peter C. Gordon, Molly Losh

**Affiliations:** 1 Roxelyn and Richard Pepper Department of Communication Sciences and Disorders, Northwestern University, Evanston, Illinois, United States of America; 2 Clinical Child Psychology Program, University of Kansas, Lawrence, Kansas, United States of America; 3 Psychology, University of South Carolina, Columbia, South Carolina, United States of America; 4 St. John’s University, Communication Sciences and Disorders, Queens, New York, United States of America; 5 Psychology, Boston University, Boston, Massachusetts, United States of America; 6 Pediatrics, Rush University Medical Center, Chicago, Illinois, United States of America; 7 Psychology and Neuroscience, University of North Carolina at Chapel Hill, Chapel Hill, North Carolina, United States of America; Harvard Medical School, UNITED STATES

## Abstract

The *FMR1* premutation (PM) is relatively common in the general population. Evidence suggests that PM carriers may exhibit subtle differences in specific cognitive and language abilities. This study examined potential mechanisms underlying such differences through the study of gaze and language coordination during a language processing task (rapid automatized naming; RAN) among female carriers of the *FMR1* PM. RAN taps a complex set of underlying neuropsychological mechanisms, with breakdowns implicating processing disruptions in fundamental skills that support higher order language and executive functions, making RAN (and analysis of gaze/language coordination during RAN) a potentially powerful paradigm for revealing the phenotypic expression of the *FMR1* PM. Forty-eight PM carriers and 56 controls completed RAN on an eye tracker, where they serially named arrays of numbers, letters, colors, and objects. Findings revealed a pattern of inefficient language processing in the PM group, including a greater number of eye fixations (namely, visual regressions) and reduced eye-voice span (i.e., the eyes’ lead over the voice) relative to controls. Differences were driven by performance in the latter half of the RAN arrays, when working memory and processing load are the greatest, implicating executive skills. RAN deficits were associated with broader social-communicative difficulties among PM carriers, and with *FMR1*-related molecular genetic variation (higher CGG repeat length, lower activation ratio, and increased levels of the fragile X mental retardation protein; FMRP). Findings contribute to an understanding of the neurocognitive profile of PM carriers and indicate specific gene-behavior associations that implicate the role of the *FMR1* gene in language-related processes.

## Introduction

The fragile X mental retardation 1 gene (*FMR1*) is associated with a constellation of cognitive-behavioral phenotypes. Residing on the long arm of the X chromosome, *FMR1* in its full mutation state (CGG repeats >200) results in fragile X syndrome (FXS), which is the most common heritable cause of intellectual disability and the leading known genetic cause of autism spectrum disorder (ASD) [1]. In its premutation (PM) state (55–200 CGG repeats), *FMR1* has also been linked to clinical and sub-clinical phenotypes ranging from subtle differences in executive functioning to more severe age-related cognitive and motor decline that occur in fragile X associated tremor/ataxia syndrome (FXTAS) [2–4]. In those with a full mutation, methylation of the *FMR1* gene shuts down production of the fragile X mental retardation protein (FMRP), which plays a critical role in brain development and function [5–9]. FMRP expression can be altered in the PM state as well [10–12]. Although molecular links with cognitive phenotypes in the PM are less clear [2, 3], the severe cognitive atypicalities associated with FXTAS appear to be related to excess production of mRNA and are likely also associated with cellular toxicity [5, 13–15], and others have reported associations between FMRP and more subtle phenotypes as well [10, 16].

Whereas FXS is quite rare (~1 per 2,500 to ~1 per 5000 individuals) [17–22], the *FMR1* PM may occur in as many as 1 in 150–390 females (and less commonly in males: 1 per 260–800), with prevalence rates varying across different ethnicities [23–27]. Given the relatively high prevalence of the *FMR1* PM among women in the general population, understanding the range of phenotypes associated with the *FMR1* PM has important public health implications, with the potential to identify clinically meaningful phenotypes among carriers, as well as informing gene-behavior relationships to better understand the genetic basis of complex traits.

Studies of PM carriers (without FXTAS, unless otherwise specified) have documented differences in a number of phenotypic domains, including mood [28, 29], social cognition [30–33], language [34–36], and executive functions, such as working memory, cognitive flexibility, and processing speed [37–42]. Subclinical autism-related personality and language features have also been observed among women with the *FMR1* PM, including elevated rates of rigid or inflexible personality traits and pragmatic (i.e., social) language violations similar to those documented among parents of individuals with autism spectrum disorder (ASD) [34, 43]. Given the high comorbidity rates of ASD and FXS (40–74% of males and 13–45% of females with FXS meet ASD criteria) [44–49], and overlap in pragmatic language profiles in particular [34, 47–50], it is possible that the *FMR1* gene might play some role in the development of ASD traits, and the language-related phenotypes of those with ASD in particular.

This study investigated language-based phenotypes associated with the *FMR1* PM through analysis of language fluency and eye movement patterns during rapid automatized naming (RAN). RAN involves serial naming of arrays of common items, either symbols (letters, numbers) and non-symbols (colors, objects), as quickly and accurately as possible (wherein symbolic conditions are typically overlearned or more automatized during early childhood) [51]. This task draws on a complex set of underlying skills, including the retrieval of lexical information, the integration of orthographic, semantic, and phonological representations, as well as a number of executive skills (e.g., working memory, processing speed, regulation of attention to avoid interference between successive items) [51–55]. A broad network of language-related brain regions, including the cerebellum, have been linked to RAN performance [51, 56, 57], many of which also appear to be impacted in the *FMR1* PM [58–60]. For instance, decreased whole brain and cerebellar volumes have been reported in the PM [58]. Relationships between *FMR1*-related variation (i.e., *FMR1* mRNA or methylation of the *FMR1* exon 1/intron 1 boundary) and membrane density in the cerebellum as well as white matter microstructures in the corpus callosum also have been reported [61].

In the single study of RAN in FXS, girls with FXS displayed slower naming during a non-symbolic condition (i.e., colors) compared to IQ-matched controls, with no differences emerging during the symbolic conditions [62]. Findings reflect how processes within RAN segregate according to those that are more automatic and over-learned (i.e., symbolic) vs. those that are less automated (i.e., non-symbolic), the latter of which require more linguistic and cognitive resources [51]. Indeed, individuals with FXS evidence significant language and executive functioning impairments [50, 63–69] that may be reflected in differences in RAN performance [62]. More specifically, prior work has indicated that the stream from perceptual encoding to articulatory processes is more susceptible to interference during non-symbolic conditions [52], with greater challenges suggesting reduced automaticity due to inefficient linguistic and executive processes [51, 52, 55, 70–72].

RAN impairments have been observed in individuals with ASD across both symbolic and non-symbolic conditions [55, 70]. Subtle RAN differences are also evident among clinically unaffected first-degree relatives of individuals with ASD, where siblings and parents have shown longer naming times, higher error rates, and eye movement patterns associated with decreased fluency [55, 70, 73]. Differences in naming and related eye movements were also found to be associated with impairments in more global social communication differences in ASD, as well as with subclinical ASD features among parents (i.e., clinical behavioral features of the BAP) [55], suggesting that differences in fundamental language processing abilities indexed by RAN can provide a window into more basic mechanistic processes that potentially underlie downstream complex social-communicative behaviors and impairments impacted in ASD and related conditions [55, 70]. Together with studies of RAN in typical development, these findings suggest that gaze and language patterns revealed through RAN might help pinpoint specific attentional, perceptual, motoric, and executive mechanisms supporting fluent language and communication [52]. Studying such processes among carriers of the *FMR1* PM, where language and executive skill differences have been reported [35, 36, 74], could therefore help to tie such complex abilities to specific genetic variation.

The current study examined the coordination of language and gaze during RAN, and potential associations with more complex language skills, to understand the mechanisms contributing to the neurocognitive sequelae associated with the *FMR1* PM. We examined naming time and error frequency in addition to gaze patterns during RAN, including eye-voice span (EVS) and fixation patterns during naming. Eye-voice span (i.e., the lead in the position of the eyes compared to the position of the item being spoken), is an index of the fluent coordination of basic components of language, reflecting pre-processing abilities (including working memory) and the fluency with which visual input (which decays quickly) [75] is transformed to phonological code that is then buffered in the phonological loop [76, 77]. The efficiency of this buffering, grapheme-to-phoneme conversion, and working memory is related to EVS length, particularly during highly automatized and familiar stimuli [72]. We also examined the total number and types of fixations during naming, including regressive and perseverative fixations. Fixation frequency is often used as a global index of cognitive effort, fluency, or processing speed, whereas the *type* of eye movement can provide additional information on the specific component abilities contributing to a processing breakdown within the orthographic encoding to articulation chain involved in RAN. Specifically, regressions (eye movements that go backwards rather than forwards) are believed to reflect slow processing speed, or a loss of mental set, an aspect of executive function that reflects challenges with maintaining on-task behaviors [78–81]. Given prior reports of differences in executive control processes and language among PM carriers, we predicted that the *FMR1* PM group would demonstrate shorter EVS and show increased visual regressions (and perseverations) in particular (given the larger executive functioning implications), with smaller differences emerging in global indices of naming time and error rates compared to controls.

Performance patterns unfolding over the course of the RAN task were also examined. Evidence suggests that task initiation processes (i.e., retrieval of item names) are distinct from the more continuous neurocognitive processes required in the latter half of the task (e.g., automated, executive processes to maintain fluency, or sustaining items in working memory). For example, Amtmann et al. [82] reported that individuals who were overall slower namers during RAN tended to show progressively longer naming times with each subsequent row, whereas individuals who were generally faster namers demonstrated a more stable naming pattern across rows. This suggests that examining patterns of naming over the course of trials, particularly when coupled with analysis of eye movement patterns, might reveal important performance differences in the nature of RAN difficulties, implicating component executive skills that have been reported to be impacted in the PM [37–42]. We therefore predicted that potential group differences in time, errors, and eye movement might stem from performance differences in the latter half of the task.

Finally, we explored associations between gaze and language coordination during RAN and broader language and executive skills. Because RAN taps fundamental language-related neuropsychological skills, reduced automaticity may result in fewer resources being available to support higher-level language skills such as pragmatic language [54], which can be impacted among PM carriers [34, 35, 43]. In line with this possibility, inefficient RAN performance in ASD has been observed to relate to impairments in pragmatic language [55]. As such, we predicted that RAN impairments, and particularly those features reflecting executive control that impact complex language processes (i.e., EVS and refixations [regressions and perseverations]), would relate to poorer pragmatic language abilities. It was also predicted that less efficient executive skills would relate to slower naming time, lower EVS, and increased visual regressions. Associations between RAN and *FMR1*-related variation (i.e., FMRP expression, CGG repeat length) were also examined.

## Materials and methods

### Participants

Participants included 48 adult females carrying the *FMR1* premutation (PM Group) and 56 controls (control group) (see Table 1 for sample characteristics). The control group in the present study also served as a parent control group in a previous study of RAN in families of individuals with ASD [55]. Families were recruited through study advertisements distributed to clinics and advocacy organizations, participant registries, and word of mouth. Inclusionary criteria for all participants included having a minimum verbal and full-scale IQ of 80, no visual impairment(s) (such as strabismus or anisometropia; participants wearing glasses for myopia or hyperopia were not excluded) or color blindness, no history of dyslexia or brain injury, no *major* psychiatric disorder (including bipolar and schizophrenia spectrum disorders), and being a fluent speaker of English. PM participants were not excluded from the study if they had a history of anxiety or depression, given the high prevalence of these disorders in the PM [44, 83]. Regardless, the Mini International Neuropsychiatric Interview (MINI) [84] was additionally administered, and showed very low rates of symptoms associated with generalized anxiety disorder in both groups (4% of PM group; 7% of controls) or a major depressive episode (2% of PM carriers; 0% of controls). As such, mood-related effects on performance was not a concern for the sample of participants included in this study. Control participants were excluded if they had a family history of ASD, dyslexia, fragile X, or language-related delays. PM status was confirmed via genetic testing (using PCR and confirmatory southern blot), or receipt of comparable testing through medical report. Mosaicism was also excluded in the PM group. Full-scale, verbal, and performance IQ were assessed using the Wechsler Abbreviated Scale of Intelligence (WASI) [85] or the Wechsler Adult Intelligence Scale (WAIS-III) [86]. Significant differences in performance IQ (PIQ) and full-scale IQ were observed between groups (*p*s < .05, see Table 1), but groups were more comparable in verbal IQ. Groups differed marginally in age, though were highly comparable in age range (Control group age 40–69 years; PM group age 35–69 years).

**Table 1:** Characteristics of the sample.

	Control group (n = 56)	PM group (n = 48)	Group Comparisons		
	M (SD)	M (SD)	t	df	*p*
Age (years)	41.34 (10.49)	44.98 (9.55)	-1.84	102	0.069
**Full IQ**	114.98 (11.15)	110.98 (8.95)	**1.99**	**102**	**0.049**
**Performance IQ**	115.18 (12.71)	109.99 (9.47)	**2.33**	**102**	**0.022**
Verbal IQ	111.17 (12.58)	109.19 (10.42)	0.87	102	0.388
CGG Repeat Length	-	87.67 (17.20)	-	-	-
Activation Ratio	-	.44 (.25)	-	-	-
Quantitative FMRP	-	.016 (.008)	-	-	-

IQ, Intelligence Quotient; CGG, Cyotisne Guanine Guanine; FMRP, Fragile-X Mental Retardation Protein

Participants were tested in a quiet laboratory space or at the participant’s residence, with comparable administration procedures across groups. All study procedures were approved by Northwestern University’s Institutional Review Board and written informed consent was obtained for all participants.

### Rapid automatized naming

#### Design and stimuli

Participants completed a Rapid Automatized Naming (RAN) task with stimuli from the Comprehensive Test of Phonological Processing (CTOPP) [87, 88]. Participants completed two runs of the task for each of the stimuli types (colors, letters, numbers, and objects—in order), for a total of eight runs. Each of the eight runs consisted of naming an array of 36 items (four rows with nine items each). The order of items in an array was pseudo-random, such that an individual item was not repeated twice consecutively. Participants were instructed to name the stimuli in each row from left to right, and to complete all rows top to bottom, as quickly and accurately as possible. Before each condition, all participants completed a practice row of nine symbols to ensure comprehension of task instructions.

Participants were seated approximately 18–24 inches away from a 17-inch TFT LCD monitor (1,280 x 1,024 resolution) (see Hogan-Brown et al. [70] for more details). A Tobii T60 eye tracker (Tobii Technology AB, Danderyd, Sweden) was used to measure gaze coordinates at a rate of 60 Hz. This device has a typical accuracy of 0.5° of visual angle. Prior to the task, eye gaze was calibrated using a standard 5-point grid. Participants were recalibrated following any large gross motor movements.

#### Data processing

Vocal Responses. The Penn Phonetics Lab Forced Aligner, an automatic and forced phonetic alignment toolkit that synchronizes phonetic transcriptions with speech signals [89] was used to mark the onset and offset of the articulation of each item. Boundaries were subsequently checked by coders who were blind to participant diagnostic status. Any errors in the marking or deviations from the expected names of the items were manually corrected by coders to reflect the participant’s actual response, including marking unexpected responses as errors [52, 55, 70].

Gaze. For eye gaze analyses, an area of interest (AOI) for each RAN item (i.e., each individual color, object, letter, or number) was operationally defined as a region extending vertically and horizontally from the middle of each item to the midpoint between each neighboring item, as has been previously done [70]. Fixations were therefore assigned to an AOI based on their spatial coordinates and consecutive fixations within the same AOI were pooled after being coded as “perseverative” fixations. Quality control steps followed previously published methods, including verifying minimum fixation duration, minimum/maximum number of fixations per run, and exclusion of specific AOIs towards the beginning and end of each run for analyses (see Nayar et al. [55] for details). On average, 3.6% of runs were excluded due to track loss for both the PM and control groups. Unpaired, two-tailed, *t*-tests revealed no significant group difference on the number of trials excluded (*t*(16) = 0.55, *p* = .59).

### Analysis procedures for RAN variables

For each variable of interest (see RAN performance and eye movement below), both runs per stimulus type (letter, number, color, object) were averaged to produce one mean variable per condition. Additionally, consistent with prior work, conditions were averaged to compare symbolic (letter/number) fluency vs. non-symbolic (object/color) fluency [51, 52, 55, 70, 71, 73]. To assess how RAN performance unfolds over the course of the task, analyses comparing performance on the first two rows and last two rows were also conducted. Following existing procedures, gaze during the first two items and final three items of the task were not included in analyses, due to greater rates of data loss and sporadic eye tracking during these items [52, 55, 70].

#### Naming performance

Overall naming time. Naming time was calculated as the time between the onset of the first articulation to the offset of the final (36th) item on each run. Row sequence analyses of naming time included naming time for the first two rows and last two rows independently.

Frequency of errors. All errors and self-corrections during RAN were totaled, including substitutions (e.g., saying “green” instead of “red”), omissions, or repetitions. Row sequence analyses were not conducted for errors given the low error rate overall.

#### Eye movement

Eye-voice span (EVS). EVS was defined as the number of items ahead the eye gaze was compared to the voice at the onset of each vocal response. EVS only included correct responses and data were omitted for two subsequent responses following errors, as eye movement patterns are often disrupted during errors due to regressions and self-corrections. Any EVS <0 or >5 for a given item was excluded from analyses, as such values likely reflect poor tracking and/or off-task behavior. EVS for row sequence analyses was calculated for the first two rows and last two rows separately.

Number of fixations overall. This was defined as the total number of fixations made during the whole run, or during the first two rows and last two rows, separately.

Refixations. Refixations were defined as the number of regressions (previously fixated AOI) and the number of perseverations (number of fixations sequentially made within the same AOI) across the entire run, or during the first and last two rows, separately.

### Assessment of pragmatic language

Given reported differences in pragmatic language among PM carriers [34, 43, 55], and evidence of links between less efficient RAN and complex social language impairments in ASD [55], pragmatic language violations were assessed to determine the extent to which RAN naming and eye movement patterns might relate to downstream language abilities. Using the Pragmatic Rating Scale [90], participants were asked a series of questions related to their life history, including questions regarding social relationships, academics, occupation, and family. This interview captures pragmatic language violations such as failure to provide the background information necessary to understand a topic and topic perseveration. Each PRS item is scored based on a three-point scale rated from 0 (trait absent), 1 (trait somewhat present), to 2 (trait definitely present). Objective coders blind to participant diagnostic status coded the interviews from video, and scores were consensus-coded. Higher scores indicate greater pragmatic language impairment.

### Assessment of executive functioning

The Behavior Rating Inventory of Executive Function—Adult Version [91] was administered to participants in the PM group. The BRIEF-A is a self-report measure composed of 75 items, which are classified into nine clinical scales (Inhibit, Self-Monitor, Plan/Organize, Shift, Initiate, Task Monitor, Emotional Control, Working Memory, and Organization of Materials). Clinical scales are additionally combined to composite constructs (Metacognition Index and Behavioral Regulation Index), which together form the Global Executive Composite (GEC). T-scores are derived for each subscale and composite index. Only the GEC T-score was used for correlational analyses in the present study.

### Molecular-genetic characterization of *FMR1*

Blood samples from the PM group were processed at the Rush University Molecular Genetics Lab to determine CGG expansion repeat length (via PCR with kits) [92] quantitative FMRP (pg/ug), and activation ratio. FMRP was derived using the Luminex Technology immunoassay [93]. Primary lymphocytes were first isolated from the blood and then stored at -80 degrees. They were then quickly thawed and spun at 4000 x g for 10 minutes, washed, lysed in the presence of protease inhibitors, rotated overnight, and spun at 16,000 x *g* for 20 minutes. Further quantification of the protein occurred using a bicinchoinic acid protein assay kit, with each lymphocyte sample assessed for duplicates. FMRP was calculated relative to a standard reference sample of a recombinant fusion protein carrying short domains of FMRP and GST-SR7. The activation ratio variable (as determined by Southern blot and described in Berry-Kravis et al., [94] is unique to female carriers of the *FMR1* premutation, as it determines the percentage of cells for which the active X chromosome contains an unaffected *FMR1* gene.

### Statistical analysis plan

#### Group comparisons

Group differences in RAN performance (i.e., RAN naming, error rate, EVS, fixations, refixations) were assessed using a 2 X 2 (group X condition) repeated measures MANOVA, with premutation status as the between-subject variable and RAN condition (symbolic or non-symbolic) as the within subject variable. Simple effect t-tests were conducted when group X condition interactions were significant, and degrees of freedom were corrected to account for non-equality of variances. Additional repeated measures MANOVAs were conducted for row sequence analyses, with follow-up planned comparison t-tests to determine if groups differed on their performance during the first two rows or last two rows. Note that errors were not included in row sequence analyses due to low error rate overall. Although significant group differences emerged in PIQ, PIQ was not consistently associated with RAN outcome variables nor is PIQ conceptually related to RAN. As such, PIQ was not added as a covariate in statistical analyses.

Significance level was set at *p* < .05 for all models, but mean differences of small to medium effects are indicated. Additionally, *p* values < .01 are noted as withstanding Bonferroni correction for multiple comparisons (to account for 5 RAN variables of interest). Since assumptions of ANOVA (e.g., normal distributions) were not met for most variables, all analyses were followed by non-parametric tests (Mann-Whitney *U*), which replicated all findings (results not shown). Associations between RAN naming and frequency of errors and gaze variables were also examined using Pearson correlations across all rows combined.

#### Associations with language, executive function, and molecular-genetic measures

Pearson correlations were conducted to examine associations between RAN performance and pragmatic language (total score) and an executive functioning index (i.e., BRIEF GEC) in the PM and control groups. All correlations were assessed for assumptions of normality by exploring Predicted Probability (P-P) plots, following simple linear regression models. Despite there being no notable deviations from normality in the residuals of RAN and phenotypic variables, to be comprehensive, Spearman rank correlation coefficients (*r*_*s*_) are additionally included.

Finally, key RAN variables were examined in relationship to *FMR1*-related variability using a series of linear multiple regression models using the lmer package [95] for R [96], and following Mailick et al. [97]. Models included RAN and genetic variables as fixed effects, with separate models conducted for each genetic variable (CGG repeat count, activation ratio, quantitative FMRP) to account for the smaller number of PM women with activation ratio data (n = 37 versus n = 39 with CGG repeat length). Subsequently, the associations between CGG and RAN variables were additionally explored accounting for activation ratio by including an interaction term with CGG repeat length and activation ratio in the models.

Given the exploratory nature of correlational analyses, these were not corrected for multiple comparisons. While we acknowledge that this increases the chance of type I error, our interpretation of the correlations was primarily based on close inspection of the overall pattern, including the size and direction of the correlations, and less reliant on inference based simply on significance testing.

## Results

**Fig 1:**
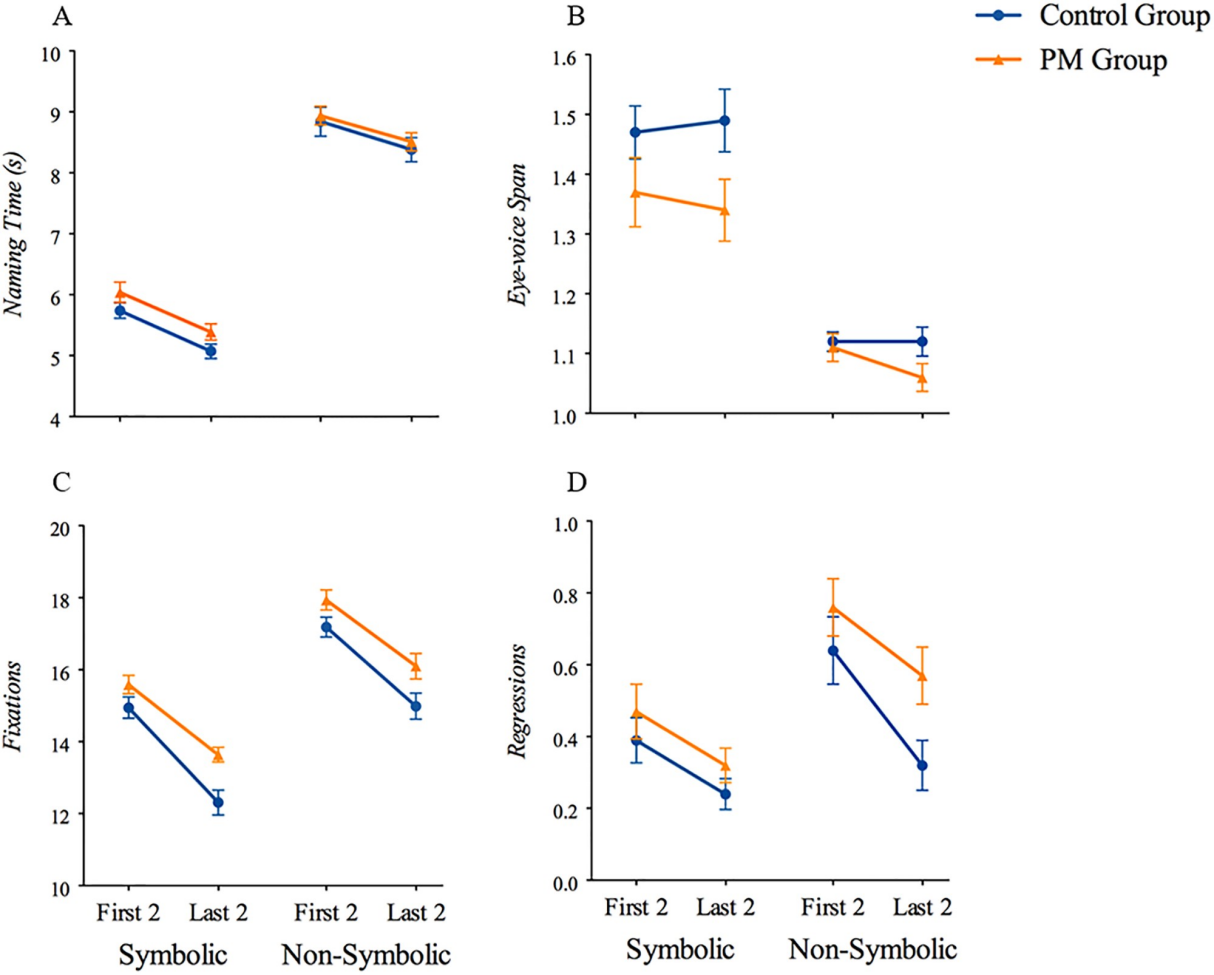
Group differences in RAN naming and gaze between PM and control groups, presented for the first 2 vs. last 2 rows of the RAN arrays. (A) Naming time, (B) Eye-voice span, (the PM group exhibited shorter EVS during the last two rows of the symbolic conditions; *p* < .05), (C) Overall number of fixations, (PM group made more fixations during the last two rows; both symbolic *p* < .01 and non-symbolic conditions *p* < .05), and (D) Regressive fixations, (PM group made more regressive fixations during the last 2 rows of the non-symbolic condition, *p* < .05).

Descriptive statistics and group comparisons are presented in Table 2 and Fig 1A (Naming performance) and Fig 1B and 1C (Eye movement during RAN), and summarized below. Row sequence analyses includes RAN performance across first 2 rows vs. last 2 rows of the RAN array.

**Table 2:** Summary of results.

	Control Group		PM Group		Main Effect of Group				Main Effect of Condition^				Group X Condition^ Interaction			
	Symbolic	Non-Symbolic	Symbolic	Non-Symbolic												
	M (SD)	M (SD)	M (SD)	M (SD)	F	df	*p*	η^2^	F	df	*p*	η^2^	F	df	*p*	η^2^
**Naming Performance**																
Overall Naming Time	13.01 (2.23)	20.37 (3.27)	13.71 (2.53)	20.92 (2.52)	1.97	1, 102	0.164	0.019	663.24	1, 102	< .0001*	0.867	0.075	1, 102	0.785	0.001
Errors	.38 (.50)	.56 (.76)	.22 (.34)	.63 (66)	0.21	1, 102	0.647	0.002	21.15	1, 102	< .0001*	0.172	2.957	1, 102	0.089	0.028
**Eye Movement**																
Eye-voice Span	1.47 (.33)	1.11 (.12)	1.35 (.37)	1.08 (.14)	2.83	1, 102	0.096	0.027	119.75	1, 102	< .0001*	0.54	2.55	1, 102	0.113	0.024
Total Fixations	27.30 (4.20)	32.67 (3.36)	29.23 (2.74)	34.04 (3.72)	8.28	1, 102	0.005*	0.075	158.62	1, 102	< .0001*	0.609	0.503	1, 102	0.48	0.005
Regressions	.63 (.62)	.93 (.93)	.79 (.69)	1.32 (.88)	4.40	1, 102	**0.038**	0.041	25.37	1, 102	< .0001*	0.199	1.89	1, 102	0.173	0.018
Perseverations	7.95 (2.50)	8.39 (4.18)	7.92 (2.43)	8.35 (2.80)	0.00	1, 102	0.951	< .0001	1.97	1, 102	0.16	0.019	0.00	1, 102	0.981	< .001

^Condition refers to symbolic (letter and number) and non-symbolic (object and color) RAN conditions.

Bold indicates significance *p* < .05;

*indicates significance following Bonferroni corrected significance value *p* ≤ .01.

### Overall RAN performance across all rows

#### Naming performance

Naming time. Both the PM and control groups exhibited longer naming times during the non-symbolic conditions. There was no main effect of group or group X condition interaction.

Frequency of errors. Both the PM and control groups committed a larger number of errors during the non-symbolic conditions. There was no main effect of group or group X condition interaction given the low error rates.

#### Eye movement during RAN

Eye-voice span (EVS). Both the PM and control groups exhibited significantly shorter EVS during the non-symbolic conditions than the symbolic conditions. No group X condition interaction was present. While there were no significant differences between groups, there was a small to medium group effect, where the PM group had shorter EVS compared to controls, particularly in the symbolic condition (planned comparisons for symbolic *t*(102) = 1.72, *p* = .09, *d* = .34 and non-symbolic *t*(102) = 1.04, *p* = .30, *d* = .20).

Number of fixations. Both the PM and control groups made a greater number of fixations during the non-symbolic conditions. No group X condition interaction was present. A main effect of group emerged in which the PM group made a significantly larger number of fixations overall (planned comparisons for symbolic *t*(95.65) = -2.82, *p* < .01, *d* = .55 and non-symbolic *t*(102) = -1.96, *p* = .05, *d* = .38).

Number of regressions. Regressions occurred significantly more often in the PM group compared to controls (though findings did not withstand Bonferroni corrections), which appeared to be specific to the non-symbolic condition (planned comparisons for symbolic *t*(102) = -1.26, *p* = .21, *d* = .25 and non-symbolic *t*(102) = -2.18, *p* < .05, *d* = .43). Both groups made more regressions during non-symbolic conditions. There was no group X condition interaction.

Number of perseverations. Both the PM and control groups made more perseverative fixations during the non-symbolic than symbolic conditions. There were no group or group X condition interactions.

### Sequential RAN performance and eye movement by rows

As depicted in Fig 1, examining performance sequentially over the course of the task revealed relatively consistent differences in eye-movement measures during the latter portion of the task for the PM group. Compared to controls, the PM group exhibited shorter EVS during the last two rows of the symbolic conditions (planned comparison for symbolic *t*(102) = 2.12, *p* < .05, *d* = .42; non-symbolic *t*(102) = 1.55, *p* = .12, *d* = .31) (Fig 1B). They also made more fixations during the last two rows (planned comparisons for symbolic *t*(87.86) = -3.30, *p* < .01, *d* = .64 and non-symbolic conditions *t*(102) = -2.17, *p* < .05, *d* = .43) (Fig 1C), which were primarily regressive fixations (planned comparison for symbolic *t*(102) = -1.28, *p* = .21, *d* = .25 and non-symbolic *t*(102) = -2.35, *p* < .05, *d* = .46) (Fig 1D). No group differences emerged over the sequence of rows for naming time, errors, or number of perseverations.

### Associations between eye movement and RAN performance across all rows

#### Naming time and eye movement

In both the PM and control groups, larger EVS during the symbolic condition was correlated with faster naming time in the same condition (*r* = -.59, *p* < .0001). Additionally, in both groups, total number of fixations was positively correlated with naming time across conditions (*r*s > .37, *p*s < .05). During the non-symbolic condition, more regressions and perseverations were associated with slower naming times (*r*s > .35, *p*s < .01) in both the PM and control groups. In the control group only, a larger number of regressions during the symbolic condition was associated with slower naming times during the same condition (*r* = .38, *p*s < .01).

#### Frequency of errors and eye movement

The number of fixations during the non-symbolic condition was positively correlated with number of errors committed by the PM group (*r* = .34, *p* < .05). In the control group, during non-symbolic conditions, number of perseverations was related to frequency of errors (*r* = .31, *p* < .05).

### Language and executive correlates of RAN ability

**Fig 2:**
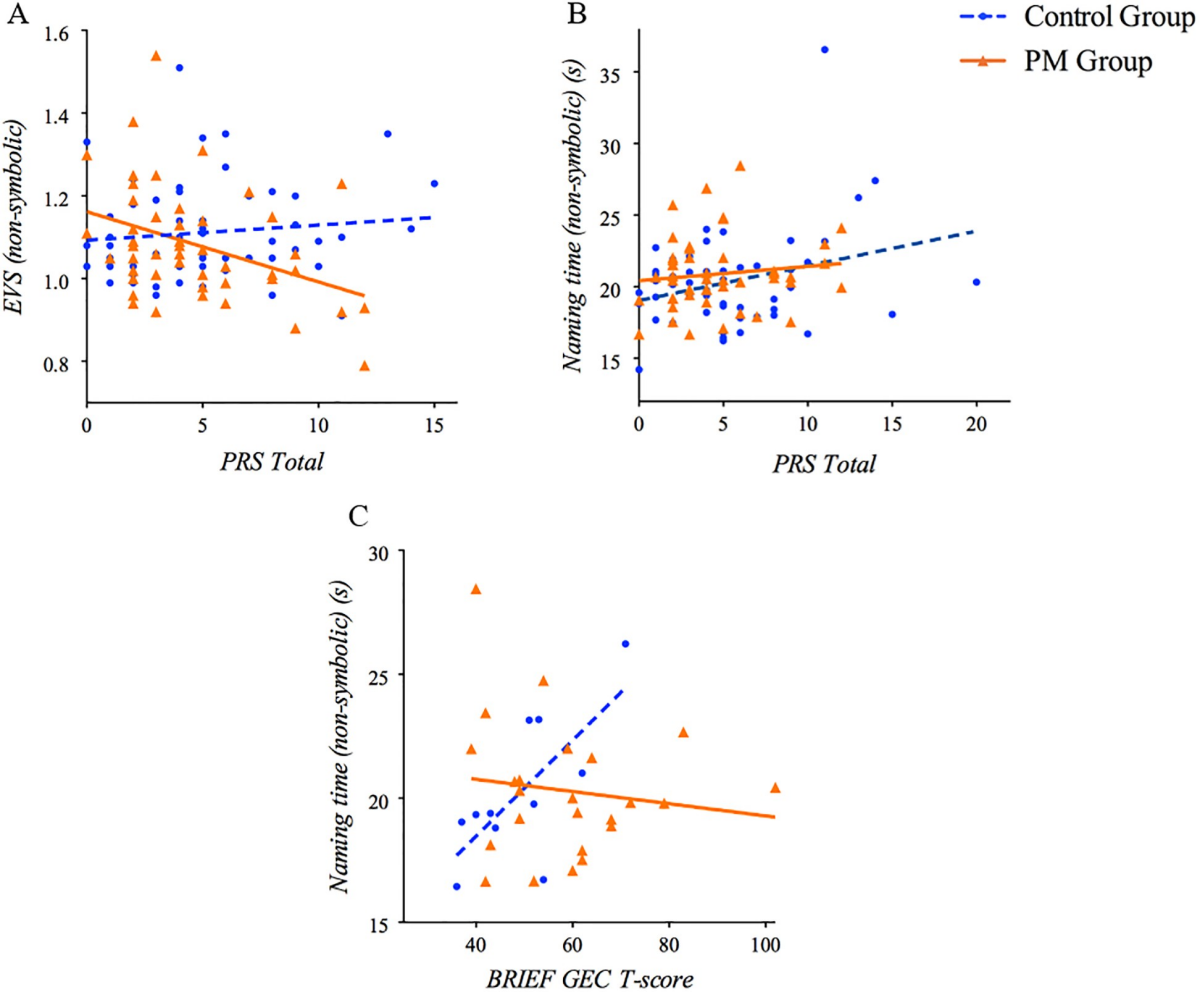
Relationships between RAN performance and gaze with clinical-behavioral correlates. (A) EVS during non-symbolic conditions associated with pragmatic language violations in the PM group (*p* < .01), (B) naming time during non-symbolic conditions associated with pragmatic language violations in the control group (*p* < .05), (C) naming time during non-symbolic conditions associated with BRIEF GEC T-scores in the control (*p* < .05) but not in the PM group.

As depicted in Fig 2A, in the PM group shorter EVS during non-symbolic conditions was significantly correlated with greater pragmatic language violations (*r* = -.38, *p* < .01; *r*_s_ = .-.36, *p* = .01). No significant associations emerged between RAN indices and BRIEF GEC T-score (*r*s < |.28|, *p*s > .18; *r*_*s*_*s* < |.30|, *p*s > .16); e.g., Fig 2C).

In controls, longer naming time during non-symbolic conditions was significantly correlated with greater pragmatic language violations (using Pearson correlations (*r* = .31, *p* < .05), but not when using Spearman correlations (*r*_*s*_ = .13; *p* = .34)) and executive functioning impairments (*r* = .71, *p* < .05; *r*_*s*_ = .63, *p* < .05) (Fig 2B and 2C).

### *FMR1*-related associations with RAN

**Fig 3:**
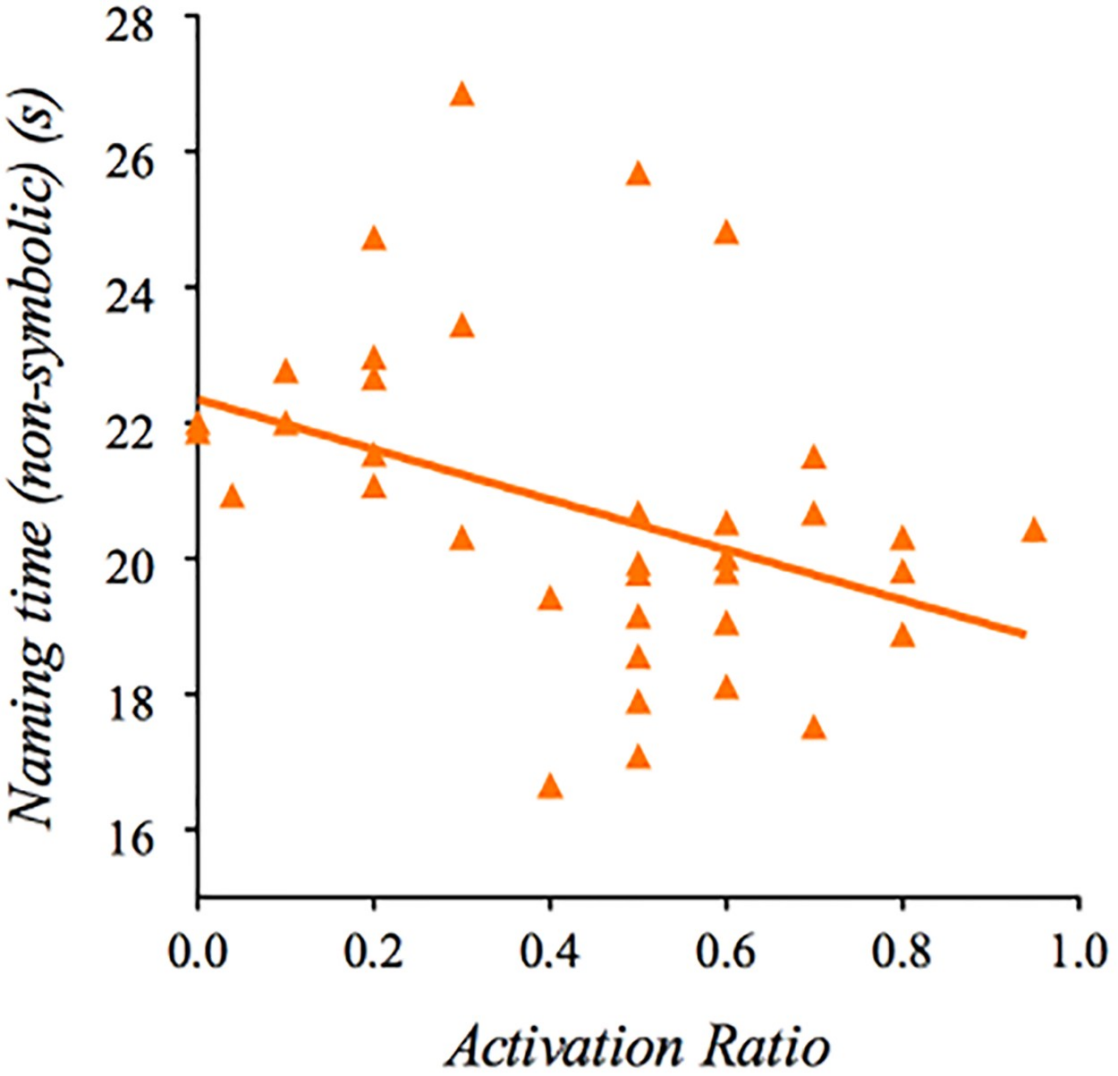
Relationship between RAN performance with molecular-genetic variation. Longer RAN naming time significantly correlated with lower activation ratios in the PM group (*ß* = -3.70, *p* < .05).

Longer naming times during the non-symbolic conditions were associated with higher FMRP levels (*ß* = 111.43, *p* < .05) and lower activation ratios (*ß* = -3.70, *p* < .05) (Fig 3) in the PM group. Additionally, number of perseverations during the symbolic conditions was significantly negatively associated with CGG repeat length (*ß* = -.07, *p* < .01).

## Discussion

This study investigated language and eye movement coordination during rapid automatized naming (RAN) among women carrying the *FMR1* premutation (PM) to further define the language-related features and underlying neurocognitive mechanisms that comprise the PM phenotype. Results revealed a complex profile of performance in the PM group, where similar naming times were observed between groups, but detailed measures of eye-voice coordination revealed more widespread differences suggestive of underlying vulnerabilities and potential compensatory strategies during RAN. Specifically, the PM group evidenced a greater number of fixations overall, and in particular, regressive fixations, compared to controls. They also demonstrated differences of small to medium effects in eye-voice span (EVS), showing less lead in the eyes compared to the voice relative to controls during the symbolic conditions (letter/number), indicative of decreased automaticity. When exploring performance patterns over the course of the RAN sequence, these differences were revealed to be driven by performance in the latter half of the stimuli, when cognitive processes are most heavily taxed, potentially implicating vulnerabilities in specific component executive processes. We also detected relationships between RAN and broader pragmatic language differences among PM carriers, as well as associations with *FMR1* variation (including both CGG repeat length and quantitative FMRP levels).

Differences observed in gaze, and gaze-language coordination in the PM group were notable given the lack of overall differences from controls in naming time, which could suggest compensatory strategies at play that mask underlying differences in language processing ability and related skills. Specifically, a greater number of fixations (particularly regressions) during RAN was observed in the PM group. Marginally shorter EVS and errors during symbolic conditions were also observed among the PM group, suggesting that female carriers are more challenged during typically automatized language tasks [51, 52, 55, 70, 73, 98]. Whereas the control group tended to lead speech by one-and-a-half items (consistent with prior studies of RAN in the general population [52, 72, 77], vocalization and gaze were more tightly coupled in the PM group. Given that EVS is a marker of fluency and coordination of linguistic and visual/attentional and working memory processes [52, 77, 99–102], reduced EVS and increased regressions (as well as marginally greater number of errors) in the PM group are suggestive of reduced efficiency in perceptual encoding, as well as a reduction in overall automaticity or fluency.

Findings that regressive fixations also predicted greater naming time may suggest that regressive eye movements reflect processing disruptions that impact language fluency in PM carriers [52]. However, it is notable that differences in eye movement were observed in the absence of any obvious differences in naming time. It could also be that the PM group does not demonstrate particular weaknesses in RAN, but that eye movement indices demonstrate a breakdown in processing that impact language fluency in other ways not reflected in naming time alone. For instance, eye movement differences may be related to underlying language processing differences that lead to verbal dysfluencies that have been reported among women with the *FMR1* PM during higher level, more complex language contexts, such as conversational interactions. Sterling and colleagues [35] identified a greater number of revisions and repetitions (both of which also characterize error patterns during RAN) during a five-minute speech sample in female carriers compared to parents of individuals with ASD, and a recent study utilizing machine learning was able to identify female carriers with a high degree of precision based on an increased number of language dysfluencies [36]. Findings that shorter EVS during RAN was associated with increased pragmatic language violations in the PM group also suggest that inefficient processing in basic RAN-related functions revealed by eye movement differences may influence downstream complex language production.

Findings that gaze differences in RAN were largely driven by the last two rows of the RAN sequence implicate more specific language processing challenges in the PM group. Specifically, whereas groups showed only subtle differences at the beginning of the trials, the PM group exhibited patterns of greater impairment, or failure to maintain performance patterns over the course of the task, revealed in eye movement indices only (i.e., EVS and regressive fixations). These results could reflect the impact of reduced sustained attention on cognitive load and flexibility in the PM group, where less efficient cognitive processes become evident when task demands for sustained attention increase. Indeed, Shelton and colleagues [42] showed that female carriers have reduced auditory processing speed and flexibility during a task that requires sustained attention and that they have lower scores on tasks of verbal fluency (rapid word generation task) compared to controls. Brain-behavior relationships detected between reduced fluency and sustained attention and brain regions including white matter tracts, cerebellum, inferior frontal operculum, anterior cingulate, and bilateral premotor cortex, indicate some overlapping areas which are implicated in the PM with and without FXTAS [58, 61, 103, 104].

Some compelling relationships between RAN and pragmatic language were observed, alongside surprising findings that RAN gaze patterns were not directly associated with executive functioning in the PM group. Specifically, the association between shorter EVS and greater pragmatic language violations may highlight how basic, component skills (pre-processing abilities and verbal working memory in particular) support more complex language functions that are impacted in the PM. On the other hand, both pragmatic language and executive functions were related to RAN performance in the control group (though, only the association between executive functions remained consistent using non-parametric Spearman correlations). It may be that different brain regions are being recruited during RAN in each group. While multiple areas of the brain are typically recruited during RAN, including executive, language, and motor networks, individuals with the PM may primarily utilize neural circuits supported by the language network, implicating reduced long-range connectivity and communication, which has previously been observed in the PM [61, 105, 106]. A similarly divergent profile has recently been reported between women with the PM and controls during an antisaccade task, where the relationship to underlying neural networks differed for these groups [59]. Further, differences in language dysfluencies but not in inhibition (a key executive skill) have also been reported among women with the PM [107].

Another potentially notable finding is that the PM group showed some similarities with patterns observed among parents of individuals with ASD. Given noted phenotypic overlap in ASD and FXS [44–49], and among PM carriers and parents of individuals with ASD [3, 34, 43], along with molecular genetic evidence of the *FMR1* gene’s interactions with many autism risk loci [108–111], findings that PM carriers showed similar RAN profiles as parents of individuals with ASD are notable. We have previously (2018) demonstrated that parents with BAP features show a greater number of refixations (including regressive fixations) during RAN, which mirrored findings in their children with ASD. Findings from the present study showed that the PM group also demonstrated more refixations than controls. Further, in addition to qualitatively similar language profiles already identified between individuals with ASD and the BAP, [34, 112–114], similar pragmatic language profiles have also been documented in subgroups of individuals with FXS [47, 48, 114] and among PM carriers [34]. Finally, previous studies have demonstrated a relationship between RAN and pragmatic language in ASD [55, 70]. Because of similar associations observed in the current study between RAN and pragmatic language (but not executive functions) in the PM group, results suggest how molecular genetic variation may play a role in language discourse in PM carriers, and also demonstrate a potential overlap with ASD-related phenotypes.

Finally, we found that poorer RAN performance (i.e., slower naming) was linked with lower activation ratios and greater FMRP levels. The correlation of poorer performance with lower activation ratios is similar to correlations seen for dysexecutive impairments in women with the PM [115]. However, the significant positive linear relationship between RAN naming and FMRP was somewhat surprising, given that the absence or reduction of FMRP causes the cognitive phenotypes in FXS, and lower FMRP levels have been hypothesized to play a role in PM phenotypes as well [2, 3, 10, 16]. While the CGG repeat length is expected to be the same in brain as in the blood samples analyzed, FMRP levels may not be concordant between blood and brain and thus may be less informative in correlation analyses with CNS-based tasks. Relatedly, the negative association between CGG repeat length and number of perseverations was somewhat surprising, particularly given prior findings of greater motor functioning deficits with increased CGG repeat length in women who carry the PM [116]. It is possible that CGG repeat length influences neural circuitry more directly linked to ataxia, such as the cerebellum [58], while the brainstem (and in particular, the reticular formation, which is responsible for generating gaze movements) may be less influenced by CGG expansion. However, Wang and colleagues [58] did report an atypical brainstem trajectory associated with the PM. Importantly, that study was conducted in males only. It will therefore be important for future studies to examine *FMR1*-related correlates of RAN with larger sample sizes, in males and females, and with larger allelic distribution (i.e., across normal, intermediate, and premutation alleles) to better understand how *FMR1* variation may relate to language processing skill and related neural circuitry.

Findings should be interpreted in light of some study limitations. Given the low error rates across both the PM and control groups, lack of differences may have been due to the low variability in the sample. It will also be important for future studies to assess RAN in male PM carriers, considering known sex differences in the PM phenotype [44]. This study included females only to reduce heterogeneity, but RAN should be explored in males as well to determine whether patterns observed here may be sex-specific. As noted previously, larger sample sizes with more variable allelic distribution will also be important considerations for future studies. Finally, given the known subtle and partially-penetrant differences of neurological functioning in female PM carriers without FXTAS, the neuropsychological differences observed in the current study suggest that atypicalities may be further reflected in underlying brain function and connectivity, and studies directly examining the relationship between language dysfluencies and neural circuitry could provide clearer insights into gene-brain-behavior relationships in the PM. Relatedly, evidence that slower naming time was associated with greater CGG repeat length is important to consider in light of evidence that increased CGG repeats may be related to motoric phenotypes [116, 117]. It is possible that vocal motor control related to the fluency of speech production or the overall motoric control over eye movements may influence performance on RAN, and this could prove an important area for further investigation.

In sum, this study’s findings enhance current understanding of the coordination between visual processing and language in the *FMR1* premutation. Particularly notable are findings that differences in gaze-language coordination, specifically as sustained attention demands increased towards the latter portion of the task, were evident without global differences in naming time. Relationships between RAN performance and pragmatic language are suggestive of how foundational processes may impact broader, more complex speech and language differences that have been previously reported in the PM. As such, findings might provide a more fine-grained understanding of the cognitive-behavior phenotype associated with the *FMR1* PM, including similarities with related conditions, such as ASD and the BAP. Quantitative and objectively measured phenotypes obtained through this study may help to provide clinically and biologically meaningful targets in future molecular-genetic studies of the impact of *FMR1*-related molecular variation on complex behavioral and cognitive-linguistic phenotypes.
